# A Simple Widespread Computer Help Improves Nutrition Support Orders and Decreases Infection Complications in Critically Ill Patients

**DOI:** 10.1371/journal.pone.0063771

**Published:** 2013-05-30

**Authors:** Mathieu Conseil, Julie Carr, Nicolas Molinari, Yannaël Coisel, Moussa Cissé, Fouad Belafia, Jean-Marc Delay, Boris Jung, Samir Jaber, Gérald Chanques

**Affiliations:** 1 Intensive Care & Anesthesiology Department, Saint Eloi Hospital, University of Montpellier Hospital, Montpellier, France; 2 Unité U1046 de l′Institut National de la Santé et de la Recherche Médicale (INSERM), University of Montpellier 1, University of Montpellier 2, Montpellier, France; 3 Department of Statistics, University of Montpellier Lapeyronie Hospital, Montpellier, France; Università Vita-Salute San Raffaele, Italy

## Abstract

**Aims:**

To assess the impact of a simple computer-based decision-support system (computer help) on the quality of nutrition support orders and patients' outcome in Intensive-Care Unit (ICU).

**Methods:**

This quality-improvement study was carried out in a 16-bed medical-surgical ICU in a French university hospital. All consecutive patients who stayed in ICU more than 10 days with non-oral feeding for more than 5 days were retrospectively included during two 12-month periods. Prescriptions of nutrition support were collected and compared to French national guidelines as a quality-improvement process. A computer help was constructed using a simple Excel-sheet (Microsoft^TM^) to guide physicians' prescriptions according to guidelines. This computer help was displayed in computers previously used for medical orders. Physicians were informed but no systematic protocol was implemented. Patients included during the first (control group) and second period (computer help group) were compared for achievement of nutrition goals and ICU outcomes.

**Results:**

The control and computer help groups respectively included 71 and 95 patients. Patients' characteristics were not significantly different between groups. In the computer help group, prescriptions achieved significantly more often 80% of nutrition goals for calorie (45% vs. 79% p<0.001) and nitrogen intake (3% vs. 37%, p<0.001). Incidence of nosocomial infections decreased significantly between the two groups (59% vs. 41%, p = 0.03). Mortality did not significantly differ between control (21%) and computer help groups (15%, p = 0.30).

**Conclusions:**

Use of a widespread inexpensive computer help is associated with significant improvements in nutrition support orders and decreased nosocomial infections in ICU patients. This computer-help is provided in electronic supplement.

## Introduction

Malnutrition may concern more than 40% of Intensive-Care-Unit (ICU) patients [1]. It is associated with increased risk of complications, length of ICU and hospital stay and hospital mortality [2], [3]. Over-feeding these patients may also lead to complications such as hepatic steatosis, hyperglycemia, hypertriglyceridemia, hypercapnia, refeeding syndrome, or increased fluid overload [4]. Existing recommendations and guidelines [5]–[7] for nutrition support of critically ill patients are often insufficiently applied [8]. In routine practice, ICU patients do not receive adequate calorie intake with estimations varying from 49 to 71% of recommended quantities [9]–[11]. The degree of ordering complexity could be one of the reasons explaining underapplication of guidelines. Thus, several strategies have been shown to improve quality of nutrition support orders such as computer-based decision-support systems [12], [13]. However, these systems used handheld devices which were not widespread and expensive. Moreover, data were limited to small cohorts of patients [12] and/or in specific area such as postoperative cardiothoracic ICU with short length of stay [13].

Compared to French national consensus recommendations [14], [15], a clinical audit of nutrition support orders in our ICU showed inadequate low energy and nitrogen delivery in respectively 51% and 89% of patients who stayed in ICU more than 10 days with non-oral feeding for more than 5 days [16]. This under-nutrition may be partly explained by physicians' lack of specific training in artificial nutrition and/or the complexity of such prescriptions requiring several calculations [17]. Indeed, this quality improvement project was started after we demonstrated the failure of our regular teaching program to educate the medical team in ordering complex artificial nutrition in ICU patients.

To improve nutrition support ordering we created an Excel spreadsheet (Office 2000, Microsoft^TM^) providing a simple tool available to all physicians on the workstations dedicated to computer-assisted ordering. This computer help is provided in electronic supplement (File S1). It helps physicians to quickly assess their nutrition support orders according to recommended goals for a given patient.

The main objective of this quality-improvement study was to determine whether a simple computer help permitted an improvement in nutrition support orders. The second objective was to determine whether improved nutrition ordering was associated with improved outcome in critically ill patients.

## Materials and Methods

### Ethics statement

The local scientific and ethics committee of Comité d'Organisation et de Gestion de l′Anesthésie Réanimation du Centre Hospitalier Universitaire de Montpellier (COGAR) approved the design of the study and written consent was waived as for quality-improvement retrospective audit aimed to implement national guidelines.

### Population

This quality-improvement study took place in the 16-bed medical-surgical ICU of the St Eloi Hospital, a 660-bed teaching and referral facility of the University Hospital of Montpellier in France. This medical-surgical ICU is specialized in gastro-intestinal diseases. All consecutive patients >18 years who stayed more than 10 days in the ICU and had fasted (defined by no oral uptake) more than 5 consecutive days were retrospectively included for a quality-improvement audit in nutrition support in two 12-month periods. There were no exclusion criteria. The control period was from June 2004 to May 2005 and the post-quality improvement intervention period was from January to December 2007.

#### Intervention aimed to implement national guidelines

**Figure 1:**
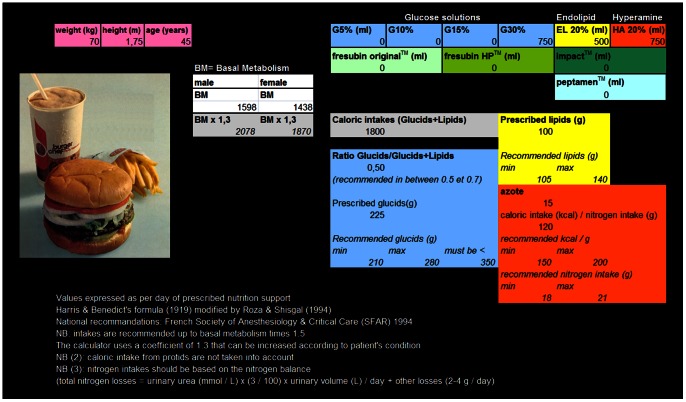
Screenshot of the Excel spreadsheet “computer-assisted prescription aid for nutrition. ” This computer help is provided in electronic supplement (File S1).

Although a qualified nutritionist is present in the ICU upon request, nutrition support was ordered routinely by the attending physician or by the resident upon supervision by the attending physician. Nutrition support was defined as enteral and/or parenteral nutrition. To help physicians for nutritional calculations, an Excel spreadsheet (Office 2000, Microsoft^TM^) was created based on French guidelines for nutrition support of critically ill patients [14], [15]. The Excel spreadsheet calculated recommended calorie and nitrogen intake for a given patient (Figure 1 and File S1). Patients' characteristics (age, height, weight and sex) are entered to estimate the basal energy expenditure according to Harris and Benedicts' equation recalculated by Roza and Shizgal [18]: 13,707× [weight in kg] +492,3× [height in cm] −6,673× [age in years] +77,607 (men) or 9,74× [weight in kg] +172,9× [height in cm] −4,737× [age in years] +667,051 (women).

Daily non nitrogen caloric needs are calculated by multiplying the basal energy expenditure thus obtained by a stress factor of 1.3 [14]. In case of obesity, defined as a Body Mass Index (BMI) (calculated as the ratio between the weight (kg) and the square of the height (m2)) >30, the weight used was the mean of the patients' real weight and ideal weight [19]. Carbohydrate calories were recommended to range between 3 and 4 g/kg/j and not to exceed 5 g/kg/j [14]. Lipid calories were recommended to range between 1.5 and 2 g/kg/j [14]. In all, it was recommended to maintain a ratio between carbohydrate calories and total non-nitrogen calories, i.e. carbohydrate and lipid calories, between 0.5 and 0.7 [14]. Daily nitrogen intake was recommended to range between 0.25 and 0.30 g/kg of ideal weight per day [14]. The physician entered into the spreadsheet the nutritional substrates he was prescribing. The corresponding energetic intake as well as each category were automatically calculated (nitrogen, lipids and glucose). The physician could compare his prescription to the recommended intake for each patient at the bedside and better adapt it according to the clinical condition. Physicians were informed about the computer help but it was not mandatory by a specific protocol. According to local guidelines, enteral nutrition was started within 24 hours after admission to ICU. The objective was to reach 100% of recommended energy and nitrogen intake within 72 hours. In case of intolerance or contraindicated enteral nutrition, total or complementary parenteral nutrition was prescribed to reach recommended intake within 72 hours. Full enteral nutrition was contraindicated throughout the study in case of gastrointestinal fistula, short small bowel, ischemic bowel, inflammatory bowel disease and occlusion or gastrointestinal intolerance (gastric residual >200 ml/6 hours despite administration of prokinetic drugs) as recommended [20]. No nutrition practices were changed during the study except the use of the computer help for nutrition ordering. Glycemic control was achieved if necessary during both study periods by an insulin drip to target a glucose concentration between 1.0 and 1.5 g/L.

#### Evaluated parameters

Baseline data collection included age, sex, height, weight, BMI, Simplified Acute Physiology Score (SAPS II) [21], Sequential Organ Failure Assessment (SOFA) score [22] and diagnosis category upon admission to the ICU (postoperative intervention of the digestive tract, liver transplant, medical digestive disease, other).

The amounts of calories and nitrogen ordered were recorded each day from the first day of stay in ICU (Day-1) until Day-15 in absence of oral feeding, or until the day oral feeding was started without any nutrition support ordering. The Duration of Nutrition Support (DNS) was calculated as the number of days when an enteral, parenteral or mix nutrition support was ordered. For each patient, daily mean non-nitrogen calorie ordering was calculated as the total ordered non-nitrogen calories, i.e. carbohydrate and lipid calories, divided by DNS and by the patient's real weight (kcal/day*kg).

The calculation was made separately for enteral and parenteral non-nitrogen ordered calories. Total non-nitrogen ordered calories were calculated as the sum of both enteral and parenteral calories. The same calculation was made for daily mean nitrogen ordering. The mean carbohydrate ratio was calculated as carbohydrate calories divided by total non-nitrogen calories for each day and then expressed as a mean over the DNS for each patient. Data were compared to the French consensus recommendations for nutrition of critically ill patients [14], [15]. The recommended non-nitrogen calories intake was 1.3 to 1.5× basal energy expenditure according to the Harris and Benedict's formula. The recommended nitrogen intake was 0.25 g/kg of ideal weight. The carbohydrate ratio was recommended to range between 50 and 70% of non-nitrogen calories. For the study purpose, mean non-nitrogen calories and nitrogen ordering was considered adequate if in between 80 and 120% of recommendations based on an energy expenditure estimated as 1.3× basal expenditure. Carbohydrate ratio was considered adequate if within the recommended norms (50 to 70% of non nitrogen calories).

Contraindications for enteral nutrition (see above) were collected from medical charts from Day-1 to Day-15, as well as any use of a nasogastric tube with postpyloric access or feeding jejunostomy.

Organ failure treatments and patients' outcomes were recorded throughout the ICU stay: renal failure as defined by the SOFA score criteria for renal dysfunction or failure [22], need for extra renal replacement therapy (RRT) and duration, administration and duration of vasopressor drugs and mechanical ventilation, length of stay and mortality in ICU. The incidence rate of ICU-acquired infection was calculated as the number of new cases of nosocomial infection diagnosed during the ICU stay divided by the number of patients included during the study period ×100.

The diagnosis of an ICU-acquired infection was defined as an infection occurring at least 48 h after admission to the ICU [23]. Pneumonia was defined as the presence of a new, progressive or persistent infiltrate on chest radiograph associated with sign and symptoms of infection including purulent tracheal secretion, leucocyte <4000/mm^3^ or >12000/mm^3^, fever, worsening oxygenation and a positive culture from bronchoalveolar lavage or protected distal sampling (>10^4^cfu/ml) or from endotracheal aspirate (>10^6^cfu/ml). Bloodstream infection was defined as a recognized pathogen cultured from 1 or more blood cultures (2 or more blood cultures for common skin contaminant). Urinary tract infection was defined as the association of a positive urine culture (>10^5^cfu/ml) and at least one sign with no other cause (fever >38°C, urgency, frequency, dysuria). Those definitions of infections acquired in the ICU had not been changed throughout the study. Data were independently and prospectively collected by the medical team as part as a regional continuous survey on ICU nosocomial infections (Coordination des Comités de Lutte contre les Infections Nosocomiales (CCLIN) Sud-Est, http://cclin-sudest.chu-lyon.fr).

#### Statistical analysis

Patients included during the first period (control group) were compared to patients included during the second period (computer help group). The primary endpoints were the proportion of patients whose calorie and nitrogen ordering were below 80% of recommended intake. Secondary endpoints were the proportion of patients whose calorie and nitrogen ordering were above 120% of recommended intake, median daily amount of calorie and nitrogen intake ordering, proportion of patients receiving enteral nutrition, carbohydrate ratio, incidence rate of ICU-acquired infections, proportion of patients receiving a RRT and the duration of RRT, length of ICU stay and mortality. The sample size was calculated based on our first audit [16] of 71 patients admitted during the first period (control group), which showed that 51% and 89% of them were prescribed less than 80% of recommended calories and nitrogen intake, respectively (primary endpoint). A 50% improvement in the quality of ordering leading to a decrease from 51% to 25% of patients prescribed adequate calories intake, with at least 71 patients included in each group, would have an alpha risk of 5% and a power of 90%. This sample size is more important than needed to show a 50% improvement in the quality of ordering leading to a decrease from 89% to 44% of patients prescribed adequate nitrogen intake.

Qualitative data are expressed as number of events (percent) and continuous data as median and interquartile range. Chi-square or Fisher's tests were used for categorical variables and non-parametric Mann-Whitney test was used for continuous variables. Changes in linear trends of targeted calories and nitrogen intake ordering over time, before and after implementation of the computer help, were estimated by interrupted time-series analysis in units of one month ordering, as previously described [24], [25]. Data were analyzed using SPSS software (SPSS Inc, Chicago, IL) and R version 2.1.1. All reported p values were two-tailed and a p value <0.05 was considered as significant.

## Results

**Figure 2:**
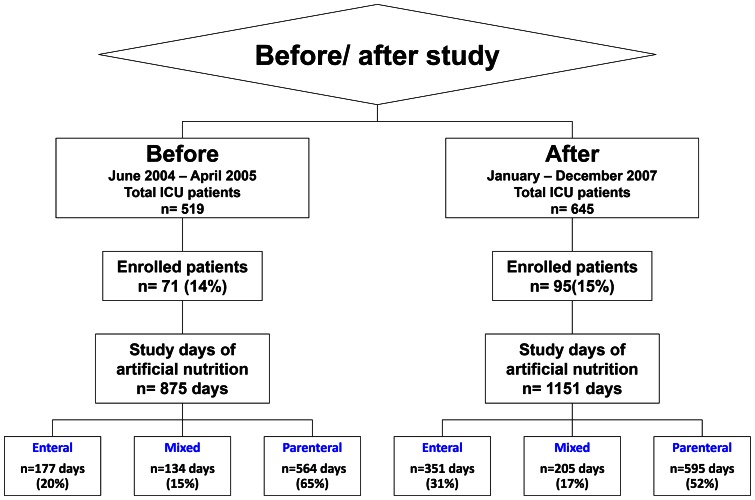
Study flow chart.

Among the 519 patients admitted to the ICU during the first period of the study, 71 met the inclusion criteria, representing 875 study days of nutrition support ordering. Among the 645 patients admitted during the second period of the study, 95 met the inclusion criteria representing 1151 days of nutrition support ordering. Figure 2 shows the flow chart of the study.

There was no significant difference between the control group and the computer-help group regarding patients' characteristics at admission to the ICU (Table 1). Patients of the entire cohort had a median BMI of 25 [22]–[28] and 65% were admitted to the ICU for an abdomen-related disease.

**Table 1:** Patients' characteristics at admission to the ICU.

	Control group	Computer-help group	*p value*
	n = 71	n = 95	
Sex (female) n (%)	32 (45)	33 (35)	0.20
Age (years)	62 [50–71]	58 [45–72]	0.47
Weight (kg)	70 [60–80]	72 [65–80]	0.47
Height (cm)	170 [163–175]	170 [160–173]	0.37
BMI (kg/m2)	25 [21]–[27]	25 [23]–[28]	0.18
SAPS II	46 [38–53]	43 [35–54]	0.56
SOFA score	8 [5]–[10]	8 [5]–[11]	0.26
Diagnosis category, n (%)			0.78
*Gastro-enterology, surgical*	29 (41)	34 (36)	
*Liver transplant*	3 (4)	6 (6)	
*Gastro-enterology, medical*	15 (21)	24 (25)	
*Other*	24 (34)	31 (33)	

Continuous variables are presented as median and interquartile range [25^th^ and 75^th^ percentile] and categorical variables as number of patients (percent). BMI: Body Mass Index. SAPS II: Simplified Acute Physiologic Score II. SOFA: Sequential Organ Failure Assessment score.

Characteristics of nutrition support from Day-1 to Day-15 are shown in table 2. The duration of nutrition support was not significantly different between the two groups (14 [10]–[15] vs. 13 [10]–[15] days). In the computer help group, the duration of exclusive enteral nutrition ordering, expressed as a proportion of artificial nutrition duration, increased while the duration of exclusive parenteral nutrition decreased. However, the difference did not reach significance (p = 0.09 and 0.06 respectively, table 2). In the whole cohort, 64% of patients had at least one full enteral nutrition contraindication and 13% had a postpyloric feeding access without any significant difference between groups (table 2).

**Table 2:** Characteristics of nutritional support during the first 15 days of ICU stay.

	Control group	Computer-help group	*p value*
	n = 71	n = 95	
Time to initiation of nutrition support (days)	1 [1]–[2]	1 [1–1]	0.07
*Duration of nutrition support (days)*	14 [10]–[15]	13 [10]–[15]	0.63
Patients with at least one day of exclusive enteral nutrition, n (%)	42 (59)	66 (69)	0.17
*Duration of enteral nutrition (days)*	6 [4]–[11]	8 [5]–[12]	0.17
Duration of exclusive enteral nutrition related to total duration of artificial nutrition (%)	0 [0–36]	14 [0–60]	0.09
Duration of exclusive parenteral nutrition related to total duration of artificial nutrition (%)	73 [33–100]	50 [12–100]	0.06
Full enteral nutrition contraindication, n (%)			
*Occlusion or enteral nutrition intolerance*	32 (45)	36 (38)	0.43
*Gastrointestinal fistula*	10 (14)	9 (9)	0.46
*Gastric tube placement contraindication*	6 (8)	11 (12)	0.61
*Inflammatory bowel disease*	3 (4)	3 (3)	1.0
*Ischemic bowel*	2 (3)	2 (2)	1.0
*At least one contraindication*	49 (69)	58 (61)	0.33
Pospyloric feeding access, n (%)	7 (10)	14 (15)	0.48
Daily non-nitrogen calorie intake ordering			
*Total (kcal/day)*	1508 [1315–1647]	1793 [1567–1956]	<0.01
*Adjusted to body weight (kcal/kg/day)*	21 [17]–[26]	24 [20]–[28]	<0.05
*Proportion of prescribed/recommended (%)*	78 [67–92]	91 [82–105]	0.01
Daily nitrogen intake ordering			
*Total (g/day)*	8 [7]–[10]	11 [9]–[13]	<0.01
*Adjusted to body weight (mg/kg/day)*	117 [87–152]	152 [112–191]	<0.01
*Proportion of prescribed/recommended (%)*	54 [43–64]	69 [56–88]	<0.01
Proportion of days with a carbohydrate ratio between 50 and 70%, n (%)	33 [16–68]	60 [33–86]	<0.01

Continuous variables are presented as median and interquartile range [25^th^ and 75^th^ percentile] and categorical variables as number of patients (percent).

**Figure 3:**
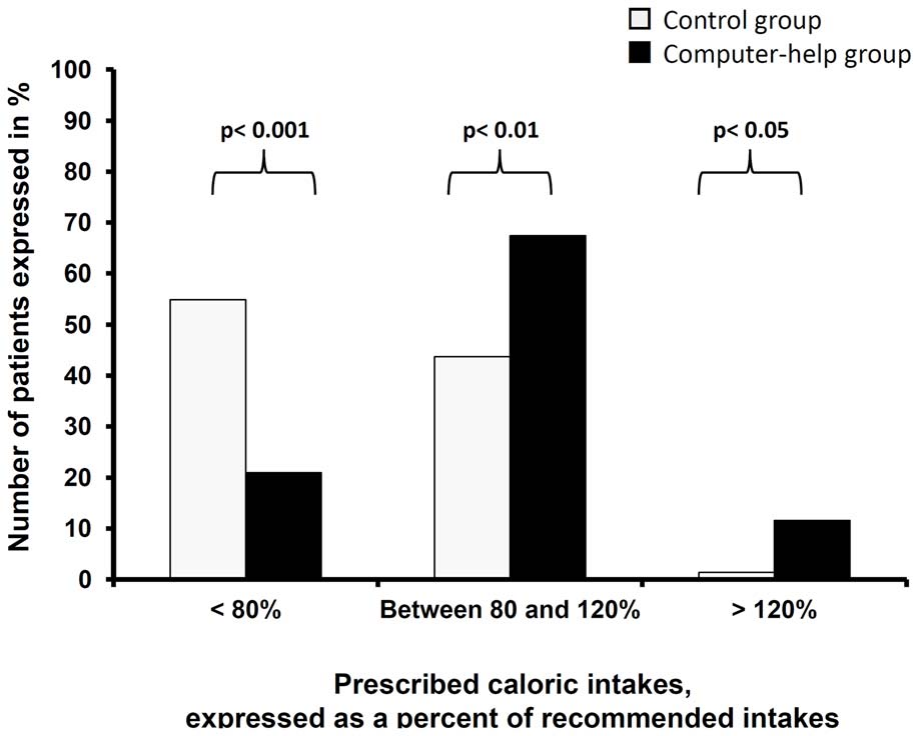
Calorie intake prescribed before and after implementation of the computer help expressed in percent of recommended goals for artificial nutrition. Introducing the computer help was associated with a significant increase in the number of patients whose prescribed calorie intake was at least 80% of recommended goals.

**Figure 4:**
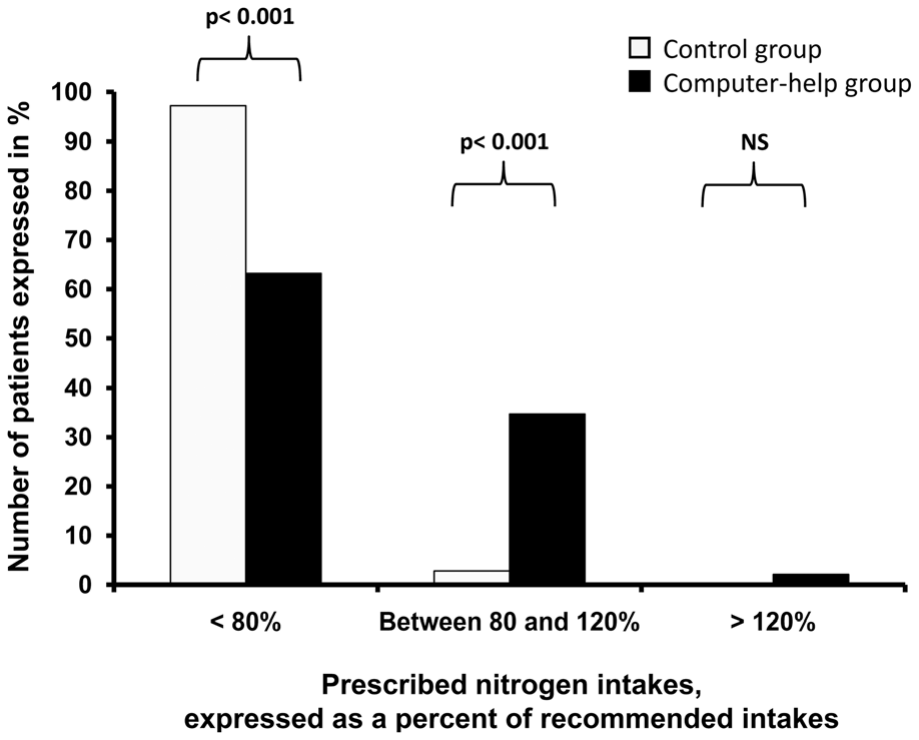
Nitrogen intake prescribed before and after implementation of the computer help expressed in percent of recommended goals for artificial nutrition. Introducing the computer help was associated with a significant increase in the number of patients whose prescribed nitrogen intake was at least 80% of recommended goals.

There was a significant increase between the two groups in calorie intake from 1508 [1315–1646] kcal/day to 1793 [1710–2058] kcal/day (p<0.01) and in nitrogen intake from 8 [7]–[10] g/day to 11 [9]–[13] g/day (p<0.01). Figures 3 and 4 show the proportion of patients with adequate and inadequate prescribed calorie and nitrogen intake. Introducing the computer help was associated with a significant increase in the number of patients prescribed at least 80% of recommended intake, both for calorie intake (45% vs. 79%, p<0.001) and for nitrogen intake (3% vs 37%, p<0.001).

The computer help was associated with a significant increase in patients prescribed more than 120% of recommended calorie intake (1% vs. 12%, p<0.05). The 11 patients who were prescribed more than 120% of recommended calorie intake in the computer help group were prescribed a median excess of 549 [478–595] calories or 34 [29]–[36] % excess in non nitrogen calorie intake.

**Figure 5:**
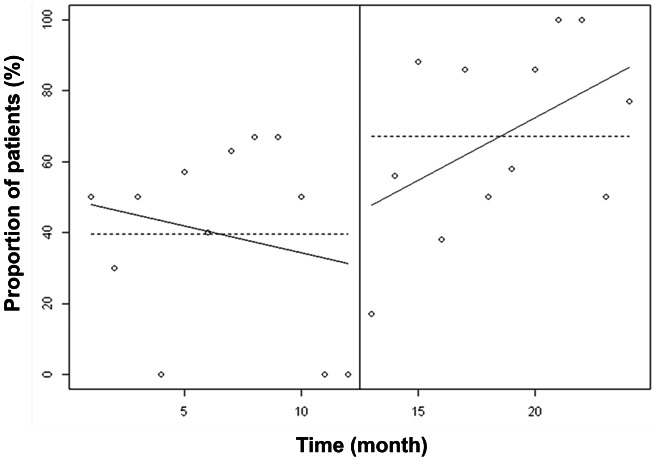
Proportion of patients with a prescribed calorie intake between 80 and 120% of recommended intake. The linear trend of adequately targeted calorie intake ordering over one year was significantly higher (p<0.0001) after than before implementation of the computer help. This suggests that there was a continuous improvement in calorie ordering over time after the implementation of the computer help.

**Figure 6:**
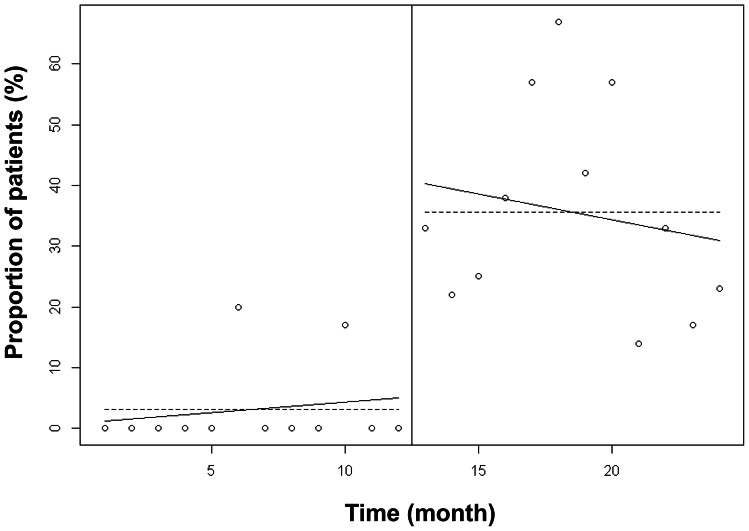
Proportion of patients with a prescribed nitrogen intake between 80 and 120% of recommended intake. The linear trend of adequately targeted nitrogen intake ordering over one year was not significantly different (p = 0.18) after than before implementation of the computer help. This suggests that improvement of nitrogen ordering did not decrease over time after the implementation of the computer help.

Nutrition ordering was improved over time with the implementation of the computer help. Figure 5 shows that the linear trend of adequately targeted calorie intake ordering over one year was significantly higher after than before implementation of the computer help. This result suggested a continuous improvement in nutrition ordering over time. The difference between linear trends of adequately targeted nitrogen intake was not significantly different between the two periods (Figure 6) suggesting that improvement in nitrogen ordering did not decrease over time after implementation of the computer help.

The proportion of orders with a glucose-lipid ratio between 50 and 70% of non protein energy as recommended was significantly higher in the computer help group (33 [16–68] % vs. 60 [33–86] %, p<0.01).

Clinical outcome at ICU discharge is showed in table 3. No significant difference in outcome was observed between the two groups regarding mortality (21% vs. 15%, p = 0.21), and length of stay in ICU (19 [14]–[28] days vs. 18 [14]–[27] days, p = 0.70). Occurrence of at least one ICU-acquired infection was significantly less frequent in the computer help group (59% vs. 41%, p = 0.03).

**Table 3:** Clinical outcome at ICU discharge.

	Control group	Computer-help group	*p value*
	n = 71	n = 95	
Renal dysfunction or failure			
*SOFA renal >1, n (%)*	29 (41)	35 (37)	0.63
*SOFA renal >2, n (%)*	14 (20)	25 (26)	0.36
*SOFA renal >3, n (%)*	11 (15)	15 (16)	1.00
*Extra renal replacement therapy, n (%)*	11 (15)	11 (12)	0.46
*Duration of extra renal replacement therapy (days)*	8 [5]–[25]	22 [10]–[30]	0.16
Vasopressive drugs, n (%)	58 (82)	69 (73)	0.17
*Duration of vasopressive drugs (days)*	5 [3]–[8]	4 [2]–[6]	<0.05
Mechanical ventilation, n (%)	67 (94)	91 (96)	0.73
*Duration of mechanical ventilation (days)*	10 [7]–[17]	11 [7]–[21]	0.83
ICU acquired infections, n (%)			
*Pneumonia*	25 (38)	23 (26)	0.17
*Bacteriemia*	15 (21)	11 (12)	0.13
*Urinary tract infection*	13 (19)	8 (8)	0.06
*At least one ICU acquired infection*	40 (59)	37 (41)	<0.05
Length of ICU stay (days)	19 [14]–[28]	18 [14]–[27]	0.70
ICU mortality, n (%)	15 (21)	14 (15)	0.31

Continuous variables are presented as median and interquartile range [25^th^ and 75^th^ percentile] and categorical variables as number of patients (percent). SOFA: Sequential Organ Failure Assessment score.

## Discussion

The main result of the present study is that a simple widespread and inexpensive computer assistance based on common sense can improve nutrition support prescription, with a 2.5-fold decrease in the proportion of patients who prescribed less than 80% of recommended calorie intake and a 1.5-fold decrease in the proportion of patients who prescribed less than 80% of recommended nitrogen intake. This improvement in nutritional support is associated with a reduction in ICU-acquired infections.

There are ample data suggesting that nutrition support recommendations and management protocols improve the achievement of target intakes, associated outcome and cost [5]–[7]. Nevertheless, practitioners' compliance is often poor [8]. Other teams have tried to improve nutritional support using protocols [26], [27] and have shown that their application led to an increased enteral intake and duration of nutrition support. However, any increase in calorie and nitrogen intake was limited or absent. Furthermore, these complex protocols required multidisciplinary training and referral to a dietician and thus were not easily applicable to all intensive care units [28].

Computer-assisted prescription has been shown to reduce workload [29] and improve prescription quality [30]. The benefit regarding nutrition support has been studied in surgical and burns patients and showed an increase in delivered calorie intake (77% of target intake versus 31% without computer assistance) [12]. Increased delivered intake was associated with less weight loss [12]. However, these computer systems were expensive and require specific software, making generalization to all ICUs difficult. Another program permitted an increase in calories and nitrogen delivery in post-surgical cardio-thoracic patients [13]. Less than 1% of the 29 patients managed with the program were prescribed less than 80% of recommended calorie intake compared to 21% of the 32 patients managed without the program. Although this program is free to access, it functions with Palm OS exploitation system which is rarely used [13].

Our system has the advantage of being very simple and does not need specific software, Excel being widespread. Furthermore the file is compatible with the Open Office series spreadsheet (Oracle Corporation, Redwood Shores, CA) which is free. The recent widespread use of smartphones by primary care team [31] will increase the availability of this simple tool since Excel spreadsheets are easy to use with several smartphones.

Enteral nutrition delivery was increased in the computer help group even if it remained lower than parenteral nutrition. Two thirds of patients admitted to our unit presented with a digestive pathology (either medical or surgical) and many had a contraindication to full enteral feeding (occlusion, short small bowel, ischemic bowel) or digestive intolerance. Several meta-analyses have compared enteral and parenteral nutrition in critically ill patients [32], [33]. None of them showed that parenteral nutrition was associated with a significantly higher mortality but it seems that it was associated with more infectious complications. Parenteral nutrition can be associated with a better proportion of prescribed nutrition actually received than enteral nutrition [34] and can help to reach target intake in case of digestive intolerance or enteral nutrition contraindications [35]. A meta-analysis even showed a lower mortality rate in patients receiving a parenteral nutrition compared to delayed enteral nutrition, but not compared to early enteral nutrition [33]. European ESPEN guidelines recommend starting parenteral nutrition within 24 to 48 hours after admission if enteral feeding cannot be tolerated or is insufficient [6]. Recently, a prospective randomized study evaluated the impact of early parenteral nutrition added to enteral nutrition compared to late parenteral nutrition in ICU [36]. Late parenteral nutrition was associated with fewer infections and shorter length of stay but mortality was similar. However in this study the majority of patients were hospitalized after cardiac surgery and the duration of ICU stay was very short (3 days vs. 18 days in our study). A randomized trial in patients expected to stay more than 3 days in the ICU showed a reduction in hospital and Day-60 mortality when parenteral nutrition was added to reach the energy target determined by calorimetry [35].

In our study an improvement in nutrition prescriptions was associated with fewer cases of ventilator acquired pneumonia and of bacteremia. ICU-acquired infections in general were significantly less frequent, which could be explained by increased calorie intake. In a study of 48 surgical ICU patients, a negative energy balance was associated with more infectious complications, greater length of stay and of mechanical ventilation, from the first week of hospitalization in ICU [3]. Another study including 138 medical patients showed that the group receiving lower energy intake presented more bacteremia than the other patients [11]. Moreover, successful enteral nutrition was associated with a reduction in infectious complications in an observational study performed in 207 medical-surgical ICU [37]. Some authors highlight a link between the introduction of a nutrition protocol and improved patient outcome: reduced mechanical ventilation [38], length of stay and hospital mortality [26]. In these studies, the increase in calorie and nitrogen intake remained limited but the nutrition support protocol was associated with more prescriptions of enteral nutrition. Other studies did not find any improvement of outcome despite increased nutritional intake [27]. Also, although total parenteral nutrition is not recommended for all ICU patients, the present computer help was designed to improve nutrient intake in selected patients such as patients admitted to our medical-surgical ICU who often suffer from gastro-intestinal disorders. It showed a significant decreased rate of nosocomial infection in this population. If this retrospective study might have benefited from other non-controlled factors associated with improvement in quality of care in the ICU, our findings are consistent with recent data from a randomized trial [39] aimed at comparing enteral nutrition alone (control group, n = 152) to a strategy achieving nutritional targets from Day-4 to Day-8 by adding parenteral nutrition if needed (intervention group, n = 153). The primary outcome was occurrence of nosocomial infection between Day-9 and Day-28. Patients in the intervention group had their nutrition managed to achieve targets by complex nutrition ordering in a way similar to our project with a similar significant decreased rate of nosocomial infections: 27 versus 38% between Day-9 and Day-28 (p<0.05) in this study; 41 versus 59% at ICU discharge (p<0.05) in the present study.

This study has several limits. First of all, with the “before/after” design, improved nutritional support could have been linked to greater awareness regarding nutrition support in the “after” group. However, this possible Hawthorne effect should be lessened because of the retrospective design of the study. Moreover, the quality of ordering improved over a large period (one year) suggesting that the computer help has been successfully implemented during the 1.5-year period of study interphase. Duration of this interphase was necessary to collect and analyze data from the first baseline phase, as well as to construct the computer help, test, adjust and implement it in a routine use. Secondly, determining caloric targets using predictive equations is imperfect compared to indirect calorimetry [40] but this method is not available in most intensive care units. We chose to use Harris and Benedicts' equation as recommended by the French consensus [14], [15]. Thirdly, we studied only nutrition as prescribed by the physician and not really received by patients. The computer help was aimed to decrease only insufficient ordering by physicians. Moreover, retrospective analysis of ordering was more reliable than attempting to estimate the patients' real intake. A significant difference between what was prescribed and what was delivered has been prospectively demonstrated in the ICU setting [9]. For this reason, and because Consensus guidelines recommend calorie intake of up to 1.5 times basal metabolism whereas the computer help used an average target of 1.3 times basal metabolism, we suppose that patients who were prescribed more calories than recommended by the computer help could have been intentionally selected by physicians to reach sufficient intake. Fourthly, the present project has began with French recommendations which have been recently updated [41]. Complex formulae such as the Harris and Benedict formula we used throughout this project are not recommended anymore contrary to simple calculations based on patients' weight only (25–30 kCal/kg). Our computer help may have increased adherence to recommendations because it might have facilitated complex calculations of patients' nutritional needs as well as effectively ordered nutritional supplies. Finally, the idea of this computer help was made up after the medical team had shown the failure of its previous teaching program aimed at ordering complex artificial nutrition according to guidelines. Consequently, the project was initiated by the medical team itself who was highly aware of the usefulness of the computer help. Further evaluation of the impact of the computer help is needed if applied in other teams apart from a comprehensive quality improvement project.

## Conclusions

Prescribed artificial nutrition intakes are often under recommended goals in critically ill patients. Complexity of prescription formulas could partly explain this observation. This study, conducted in a medical-surgical ICU with an orientation towards digestive pathologies, showed that a simple, widespread and inexpensive program could dramatically increase prescribed patients' energy and protein intake and improve the application of recommendations for artificial nutrition of critically ill patients. This improvement of prescriptions was associated with a decrease in infectious complications.

## Supporting Information

File S1 **Computer-assisted prescription aid for nutrition.** Excel spreadsheet (Office 2000, Microsoft^TM^) created to help physicians for nutritional calculations.
(XLS)
